# Proteome-wide analysis of hydrogen peroxide-induced protein carbonylation in *Arabidopsis thaliana*

**DOI:** 10.3389/fpls.2022.1049681

**Published:** 2022-12-05

**Authors:** Georges Yannick Fangue-Yapseu, Adesola Julius Tola, Tagnon D. Missihoun

**Affiliations:** Groupe de Recherche et Biologie Végétale (GRBV), Department of Chemistry, Biochemistry and Physics, Université du Québec à Trois-Rivières, Trois-Rivières, QC, Canada

**Keywords:** cyanine hydrazide, hydrogen peroxide, metal-catalyzed oxidation, plant defense response, protein carbonylation, reactive electrophile species

## Abstract

**Introduction:**

Protein carbonylation is a non-enzymatic and irreversible post-translational modification that occurs naturally in living organisms under the direct or indirect effect of reactive oxygen species (ROS). In animals, signaling pathways involving numerous carbonylated proteins have been identified, highlighting the dual role of these molecules in ROS signal transduction. In plants, studies on phytohormone signaling (auxin, methyl jasmonate, abscisic acid) have shown that reactive carbonyl species (RCS: acrolein, malondialdehyde, 4-hydroxynonenal, etc.), derived from the action of ROS on lipids, play important roles in secondary root formation and stomatal closure. However, the carbonylated proteins involved in these signaling pathways remain to be identified.

**Methods:**

In this study, we analyzed proteins responsive to carbonylation by exogenous hydrogen peroxide (H2O2) by profiling the carbonyl proteome extracted from Arabidopsis thaliana leaves after H2O2 treatment. Carbonylated proteins were enriched at the peptide level and analyzed by liquid chromatography coupled to tandem mass spectrometry (LC-MS/MS).

**Results and discussion:**

We identified 35 and 39 uniquely carbonylated proteins in the untreated and the H2O2-treated plant samples, respectively. In comparison to the control treatment, gene ontology enrichment analysis revealed that most of the carbonylated proteins identified in the H2O2-treated plant samples are related to sulfate adenylyl transferases and amidophosphoribosyl transferases involved in the immune system response, defense response, and external stimulus-response. These results indicated that exogenous H2O2 caused a change in the pattern of protein carbonylation in A. thaliana leaves. Protein carbonylation may thus influence the plant transcriptome and metabolism in response to H2O2 and ROS-triggering external stimuli.

## Introduction

Protein carbonylation is a non-enzymatic and irreversible post-translational modification linked to reactive oxygen species (ROS; superoxide anion radical: $0_{2}^{\mathrm{•-}}$ ; hydrogen peroxide: H_2_O_2_; hydroxyl radical: OH^•^). Protein carbonylation can be beneficial or harmful to the cell or the organism (Fedorova et al., 2014; Reichmann et al., 2018; Tola et al., 2021). It interferes with the structural and functional properties of proteins, thus affecting their molecular and cellular interactions (Jaisson et al., 2017).

In animals and humans, protein carbonylation is considered a proven marker of oxidative stress (Fritz and Petersen, 2011; Fedorova et al., 2014) and reflects ROS-induced cell damage (Dalle-Donne et al., 2006). Signaling pathways involving the carbonylation of specific proteins have also been identified. For example, the carbonylation of the Keap1 protein by 2-hydroxynonenal (HNE) on its critical cysteine residues (Cys273 and Cys288) has the effect of releasing the nuclear transcription factor NRF2 (nuclear factor erythroid-2-related factor 2) which controls the expression of antioxidant and cytoprotective genes *via* the regulatory sequence called the antioxidant response element (Curtis et al., 2012). Similarly, the carbonylation of the critical cysteine residues (Cys32 and Cys35) of the mammalian thioredoxin 1 (Trx1) by HNE was found to release the protein Apoptosis Signal-Regulating Kinase 1 (ASK1) the phosphorylation of which induces a reaction cascade resulting in the activation of pro-inflammatory genes (Curtis et al., 2012). The importance of ROS-induced protein carbonylation remains poorly understood in plants (Tola et al., 2021). However, studies showed that RCS participate in the mechanism of hormone signal transduction in plants. For example, (Biswas et al., 2019) showed that in auxin-stimulated *Arabidopsis thaliana* roots, the production of ROS *via* the hormonal activation of NADPH oxidases caused the levels of the reactive carbonyl species (RCS) acrolein, 4-hydroxynonenal (HNE) and crotonaldehyde to increase prior to the formation of lateral roots. Accordingly, the addition of carnosine, a carbonyl scavenger, suppresses auxin-induced lateral root formation. Similarly, RCS were found to accumulate prior to the closure of stomata and following the production of ROS induced by the treatment of leaves with abscisic acid (ABA) and methyl-jasmonate (MeJA) (Islam et al., 2016; Islam et al., 2020). The scavenging of the RCS inhibited the closure of stomata upon ABA and MeJA. Recently, we identified a set of proteins that is specifically carbonylated in the leaves of *Arabidopsis thaliana* in response to ABA (Jaballi and Missihoun, 2022). These studies established that the carbonylation of some plant proteins by the ROS is involved in the formation of secondary roots, stomatal closure, and signaling pathways of ABA, MeJA, and auxins.

ROS cause protein carbonylation by two general mechanisms forming either primary or secondary carbonylation products. The primary products are obtained by direct attack of the side chains of the residues of Lys, Arg, Pro and Thr by ROS, or by metal-catalyzed oxidation in the presence of H_2_O_2_ (Wong et al., 2008; Fedorova, 2017; Ciacka et al., 2020; Rodríguez-García et al., 2020; Tola et al., 2021). The secondary products result from the addition of either the RCS including malondialdehyde (MDA) and the α, β-unsaturated aldehydes and ketones (HNE, 4-oxo-2-nonenal, acrolein, etc.) or advanced glycation end products on Cys, His and Lys (Wong et al., 2008; Ciacka et al., 2020). Since the RCS derive from the peroxidation of membrane lipids by ROS, H_2_O_2_ is likely a major trigger of protein carbonylation *in vivo*. In this study, we examined this hypothesis by profiling the carbonylated proteome in response to exogenous H_2_O_2_. Our results showed that number of *A. thaliana* leaf proteins mostly related to the photosynthesis, plant defense response, and sulfur metabolism were differentially targeted for carbonylation by exogenous H_2_O_2_.

## Material and methods

### Plants and growth conditions

Twenty-one (21) days old seedlings of *A. thaliana* ecotype Colombia-0 (Col-0) were used in this study. The seeds were spread on moist sterile soil and stratified for 48 h at 4°C before being transferred to a Conviron growth chamber for germination (light/dark cycle: 12/12 h; white light: 80 µmol/m²/s; day/night temperature: 21/18°C; relative humidity: 60%). Seven days after the emergence of the seedlings, the seedlings were transplanted into honeycomb trays and their growth was monitored for 14 days.

### Treatments

For treatment, the seedlings were sprayed with 50 mL of 1, 5, 10, 20 and 50 mM H_2_O_2_ solutions (T1, T5, T10, T20, and T50, respectively) prepared from a stock solution of 30% H_2_O_2_ (Fischer Scientific). The seedlings of the control batch were treated with distilled water (T0). One hour after the treatment, the different samples for each treatment were made by combining the leaves harvested from a total of six seedlings. These samples were instantly frozen in liquid nitrogen and then stored at -80°C for subsequent analyses.

### Protein extraction and labeling

For protein extraction and labeling, we used the method of Jaballi and Missihoun (2022) with some modifications. Briefly, the proteins were extracted from the plant powder at 4°C in 300 μL of a lysis buffer (25 mM Tris-HCl, pH 8; 0.1% v/v Triton X-100; 50 mM DTT; 10 mM EDTA; Sigma protease inhibitor) by centrifugation at 15,000 g for 10 min. The protein extracts of the H_2_O_2_-treated plants were immediately labeled with the fluorescent cyanine 7.5 hydrazide probe (Cy7.5-Hz; excitation/emission: 788/808 nm), while those of the control samples were labeled either with Cy7.5-Hz or cyanine 5.5 hydrazide (Cy5.5-Hz; excitation/emission: 684/710 nm). The probes were purchased from Lumiprobe (USA) and dissolved in dimethyl sulfoxide to 10 mM stock solution. The labeling was carried out in the dark and at 23°C in a reaction buffer (0.1 M sodium acetate, pH 6.8; 0.1% SDS; 1 mM EDTA) for 1 h at 500 rpm. The reaction was stopped by adding sodium cyanoborohydride and saline phosphate buffer (PBS, pH 7) to the reaction medium, followed by incubation for 15 min at room temperature. Then, the labeled proteins were precipitated in one volume of 20% (v/v) trichloroacetic acid, washed with an ethanol/ethyl acetate solution (1:1), air-dried, and finally re-suspended in the lysis buffer.

### In-gel analysis of carbonylated proteins

The carbonylated proteome was analyzed by 12.5% (w/v) sodium dodecyl sulfate polyacrylamide-gel electrophoresis (Laemmli, 1970). Ten micrograms (10 µg) of labeled proteins were loaded into the gel. After separation of carbonyl proteins, the gel was fixed with Azure A solution, then stained for total proteins with Azure red solution (excitation/emission: 520/610 nm) in Azure B staining buffer according to the manufacturer’s recommendations (Azure Biosystems, USA). The gel image was then captured using the multiplex scanning method on the Azure Biosystems scanner (software version 1.2.1228.0) at different wavelengths (Cy5.5-Hz: 658 nm; Cy7.5-Hz: 784 nm) for the detection of carbonylated proteins. The red color and green color have been assigned to Cy5.5-Hz and Cy7.5-Hz, respectively. Total proteins were detected by gel scanning at 520 nm. The gray color was attributed to total proteins. Band intensity was measured with Azure Spot software (version 2.1097). To obtain a quantitative measure of the carbonylated proteins in each sample, we proceeded as described by Jaballi and Missihoun (2022). The values of the fluorescence intensity for each gel lane were exported into Excel for further processing. The data were normalized by dividing the fluorescence intensity value of Cy5.5 or Cy7.5-labeled samples with that of Azure Red corresponding to the proteins in the sample. The arithmetic mean and the standard deviation were calculated for each sample.

### Protein carbonyl enrichment for LC-MS/MS analysis

To identify the carbonylated proteins contained in the different samples by liquid chromatography coupled to tandem mass spectrometry (LC-MS/MS), the carbonylated proteins were enriched at the peptide level (Matamoros et al., 2018). A hundred micrograms of proteins were used as a starting material. The lysis buffer was first exchanged into 100 μL of 50 mM ammonium bicarbonate (ABC, 50 mM) by using a centrifugal device (Amicon Ultra-0.5; Millipore Sigma). The protein sample was then added with 11 μL of 10% deoxycholate and 1.5 μL of Tris-2-carboxyethylphosphine (TCEP-HCl, 500 mM) to reduce the disulphide bridges at 60°C and 500 rpm for 30 min. For alkylation, 3.5 µL of 300 mM 2-iodoacetamide (IAA) was added followed by incubation in the dark at 37°C for 30 min at 550 rpm. Finally, the proteins were digested by adding 10 µL of a 0.2 µg/µL trypsin solution in the dark at 30°C for 16 h at 500 rpm. This reaction was stopped with 1.2 µL of formic acid.

The carbonylated peptides were labeled with an aldehyde reactive probe (ARP; Dojindo Laboratories). ARP is a biotinylated hydroxylamine derivative, N’-aminooxy-methylcarbonylhydrazino-D-biotin, used for the derivatization, enrichment, and mass spectrometric characterization of the carbonylated proteins (Chavez et al., 2006). The hydroxylamine group of ARP specifically forms aldoxime/ketoxime derivatives with the aldehyde/keto groups present in carbonylated proteins, which are sufficiently stable for the subsequent analysis by LC-MS/MS. The use of ARP has been described as a method of choice in several studies to probe the carbonylated proteins and to facilitate the affinity purification and enrichment of the peptides with carbonylated residues (Bollineni et al., 2013; Fedorova, 2017; Matamoros et al., 2018). For this, one volume of 10 mM ARP was added to the peptides and the mixture was incubated in the dark at 27°C for 2 h at 700 rpm. The unbound ARP molecules were then removed from the medium by the solid phase separation method using the C18 column according to the manufacturer’s recommendations (Agilent Technologies). After elution and vacuum dehydration in SpeedVac, the peptides were resuspended in 200 µL of PBS, then the carbonylated peptides were separated from the non-carbonylated peptides by affinity chromatography using an avidin slurry agarose column according to the manufacturer’s recommendations (Thermo Fisher Scientific). The carbonylated peptides trapped by the avidin resin were eluted 5 times with 150 µL of a 0.1 M glycine solution, pH 2.8 at room temperature. The different fractions were neutralized with a 1 M Tris-HCl solution, pH 8, combined, partially dehydrated to a volume between 100-150 µL, desalted using the C18 column (Agilent Technologies), and finally completely dehydrated with a SpeedVac. The samples were stored at -80°C for subsequent analysis by mass spectrometry.

### LC-MS/MS analysis and database searching

Mass spectrometry analyses and database searching were performed by the Proteomics Platform of the CHU de Québec Research Center (Quebec, Qc, Canada), as reported previously (Jaballi and Missihoun, 2022). The desalted peptides were analyzed by nanoLC/MSMS using a Dionex UltiMate 3000 nanoRSLC chromatography system (Thermo Fisher Scientific, USA) connected to an Orbitrap Fusion mass spectrometer (Thermo Fisher Scientific, San Jose, CA, USA). Peptides were trapped at 20 μl/min in loading solvent (2% acetonitrile, 0.05% TFA) on a 5mm x 300 μm C18 pepmap cartridge pre-column (Thermo Fisher Scientific/Dionex Softron GmbH, Germering, Germany) for 5 minutes. Then, the pre-column was switched online with a Pepmap Acclaim column (ThermoFisher) 50 cm x 75 µm internal diameter separation column and the peptides were eluted with a linear gradient from 5-40% eluent B (A: 0.1% formic acid, B: 80% acetonitrile, 0.1% formic acid) within 90 minutes, at 300 nL/min for a total run time of 2 hours. Mass spectra were acquired in a data-dependent acquisition mode using Thermo XCalibur software version 4.3.73.11. Full scan mass spectra (350 to 1800m/z) were acquired in the orbitrap using an AGC target of 4e5, a maximum injection time of 50 ms and a resolution of 120 000. Internal calibration using lock mass on the m/z 445.12003 siloxane ion was used. Each MS scan was followed by MSMS fragmentation of the most intense ions for a total cycle time of 3 seconds (top speed mode). The selected ions were isolated using the quadrupole analyzer in a window of 1.6 m/z and fragmented by Higher energy Collision-induced Dissociation (HCD) with 35% collision energy. The linear ion trap detected the resulting fragments at a rapid scan rate with an AGC target of 1e4 and a maximum injection time of 50 msec. Dynamic exclusion of previously fragmented peptides was set for a period of 20 seconds and a tolerance of 10 ppm.

MGF peak list files were created using Proteome Discoverer 2.3 software (Thermo). MGF sample files were then analyzed using Mascot (Matrix Science, London, UK; version 2.5.1). Mascot was set up to search a contaminant database and Uniprot Reference *Arabidopsis thaliana* database (September 2020; 39449 entries) assuming digestion with trypsin. Mascot was searched with a fragment ion mass tolerance of 0.60 Da and a parent ion tolerance of 10.0 ppm. Carbamidomethyl of cysteine was specified in Mascot as a fixed modification. Deamidation of asparagine and glutamine and oxidation of methionine were specified in Mascot as variable modifications. Two missed cleavages were allowed. Scaffold (version Scaffold_5.0.1, Proteome Software Inc., Portland, OR) was used to validate MS/MS-based peptide and protein identifications. A false discovery rate of 1% was used for peptides and proteins. Proteins that contained similar peptides and could not be differentiated based on MS/MS analysis alone were grouped to satisfy the principles of parsimony.

### Determination of malondialdehyde content and catalase enzymatic activity

Extraction and assay of MDA were based on the method of Heath and Packer (1968) and performed as described before (Missihoun et al., 2011). Briefly, 100 mg of leaves were ground in 1 mL of 5% trichloroacetic acid (TCA) containing 1.25% glycerol. After centrifugation at 15,000 g for 10 min at 4°C, 1 mL of supernatant was added to 1 mL of 0.67% thiobarbituric acid (TBA) and the mixture was heated to 100°C for 30 min, followed by rapid cooling in ice. The specific absorbance of the TBA-MDA adduct was determined by taking the difference between the absorbance value read at 532 nm and that read at 600 nm. The amount of MDA (µmol/g FW) was calculated with reference to the Beer-Lambert law (Ɛ =155 nM-1 cm-1). Total protein catalase activity was measured by the hydrogen peroxide reduction method (Chance and Maehly, 1955).

## Results

### SDS-PAGE analysis of H_2_O_2_-induced protein carbonylation in the *Arabidopsis thaliana* plants

**Figure 1:**
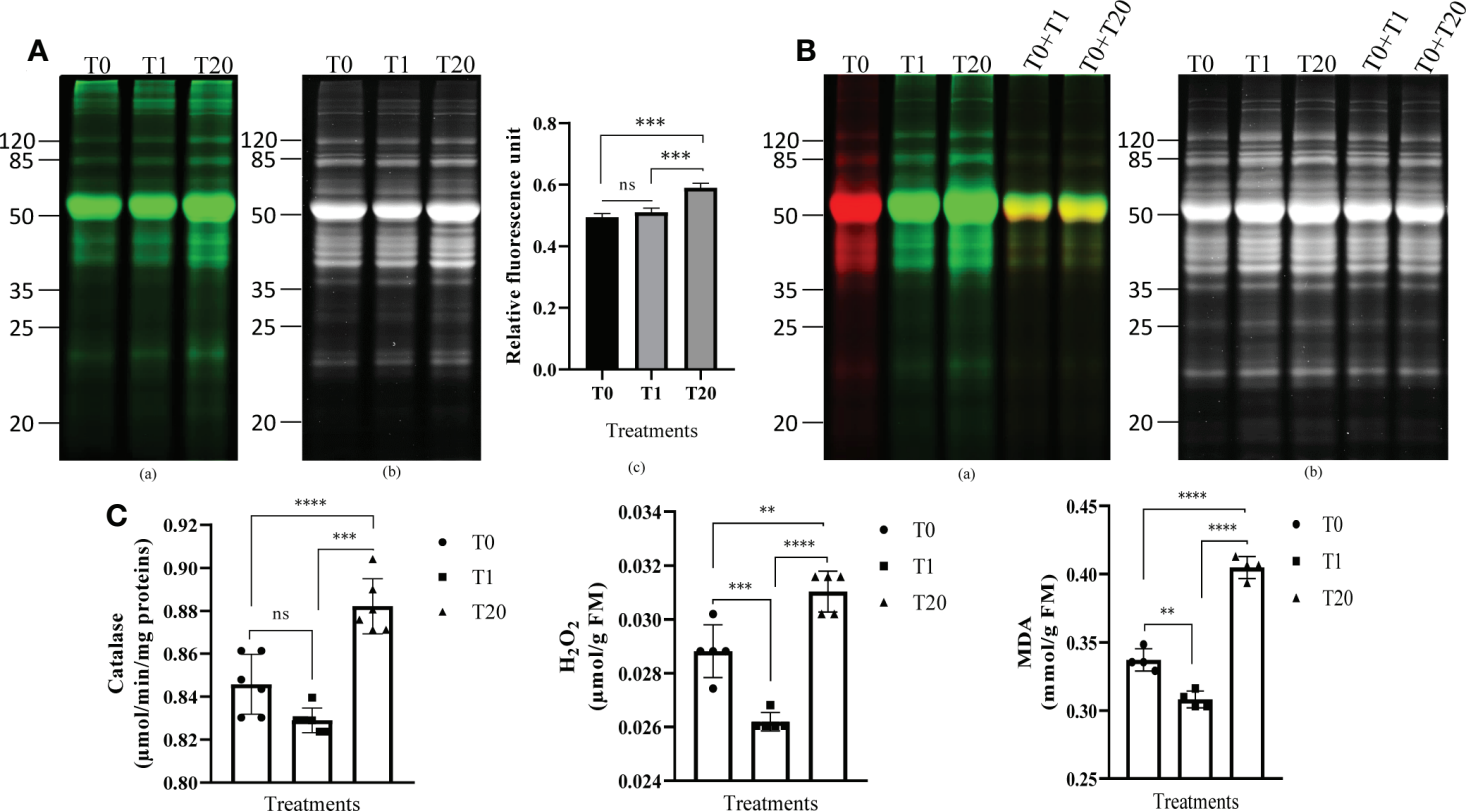
Exogenous H_2_O_2_ modulates protein carbonylation in *A. thaliana* plant leaves. **(A)** Representative gel pictures for carbonylated proteins **(a)** and the total proteins stained with AzureRed **(b)** are shown. A total of 10 µg of proteins labeled with the carbonyl-reactive probes Cy7.5-Hz probe was loaded in each lane. T0: control, distilled water; T1: H_2_O_2_ at 1 mM; T20: H_2_O_2_ at 20 mM. The bar plot **(c)** represents relative fluorescence intensity of carbonylated proteins measured from the gel picture **(a)** by using the Azure biosystems software. Results are from at least three independent experiments and represent means ± SEM (n=3). Asterisks denote statistical significance at p < 0.001, one-way ANOVA, Tukey *post hoc* test. ns: not significant. **(B)** Two probes-based gel comparison of carbonylated protein profiles. Representative gel pictures for the carbonylated proteins **(a)** and the total proteins stained with AzureRed **(b)** are shown. T0: control, distilled water; T1: H_2_O_2_ at 1 mM; T20: H_2_O_2_ at 20 mM. Carbonylated proteins in the control sample were labeled with Cy5.5-Hz probe, and those in the H_2_O_2_-treated plant samples were labeled with Cy7.5-Hz probe. Lanes T0+T1, and T0+T20 combine each equal amount (5 µg) of the Cy5.5-Hz-labeled control and Cy7.5-Hz-labeled H_2_O_2_-treated samples. **(C)** Catalase enzyme activity **(a)**, H_2_O_2_ level **(b)**, and MDA **(c)** in leaves of 21 days-old seedlings. Results are from at least three independent experiments and represent means ± SEM (n=3). Two, three, and four asterisks denote statistical significance at p < 0.01, p < 0.001, and p < 0.0001, respectively; one-way ANOVA, Tukey *post hoc* test. ns: not significant.

To assess the effect of exogenous H_2_O_2_ on protein carbonylation, 21-day-old seedlings were sprayed with various concentrations of H_2_O_2_ solutions. The seedlings of the control treatment were treated with distilled water. After 1 h of incubation, the proteins were extracted, and the carbonylated proteins were labeled with cyanine hydrazide (Cy7.5-Hz) probe prior SDS-PAGE. We found that a treatment with 20 mM H_2_O_2_ triggered an increase of the relative intensity of the fluorescent probe, thus reflecting a higher level of carbonylated proteins compared to a treatment with 1 mM H_2_O_2_ or to the untreated plants (**Figures 1A**, **S1**). To better assess the effect of exogenous H_2_O_2_ on the carbonylation of proteins, we mixed 5 µg of proteins derived from the untreated plants and labeled with Cy5.5-Hz (red fluorescence) with 5 µg of Cy7.5-Hz-labeled proteins (green fluorescence) derived from the plants treated with H_2_O_2_ and then analyzed the mixture by SDS-Page. We found that the green fluorescence was prominent for several protein bands indicating a high level of protein carbonylation induced by the H_2_O_2_ treatment (**Figures 1B**, **S2**). Consistently with the increased of carbonylated proteins, the treatment with 20 mM H_2_O_2_ significantly increased the contents of malondialdehyde, (MDA) (p < 0.0001), a marker of lipid peroxidation, the contents of H_2_O_2_ (p < 0.01), and the catalase activity (p < 0.0001) compared to the plants treated with 1 mM and the untreated plants (**Figure 1C**). Additional tests revealed that the levels of carbonylated proteins in the plants treated with 5 and 10 mM H_2_O_2_ were alike the levels seen in the plants treated with 1 mM H_2_O_2_ whereas the level of carbonylated proteins in the plants treated with 50 mM H_2_O_2_ was similar to that found in the plants treated with 20 mM H_2_O_2_ (**Figure S3**). These results indicated that a concentration of 20 mM H_2_O_2_ would be optimal to profile the proteins targeted for carbonylation by H_2_O_2_. Subsequent experiments were then conducted with 1 mM and 20 mM H_2_O_2_ in comparison with the untreated plants; 1 mM being the smallest treatment that did not induce the increase in of protein carbonylation signal, and 20 mM being the treatment from which this signal markedly increased.

### Detection of H_2_O_2_-induced carbonylated proteins by LC-MS/MS

**Figure 2:**
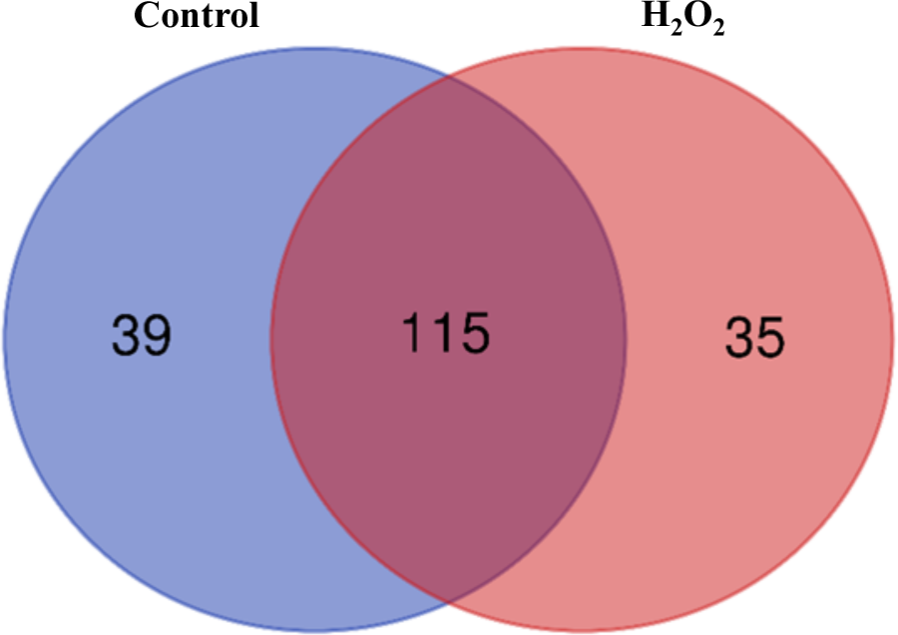
A Venn diagram of the carbonylated proteins in the control plant samples and the H_2_O_2_-treated plant samples. The diagram was generated from the carbonylated proteins identified in all three biological replicates of the samples.

To identify the carbonylated proteome in response to exogenous H_2_O_2_, the protein extracts derived from the untreated plants and the plants subjected to 20 mM H_2_O_2_ were treated with the aldehyde reactive probe (ARP) to label the carbonylated proteins, which were subsequently pulled down with an avidin matrix and then analyzed by LC-MS/MS. We identified 311 and 293 carbonylated proteins for the three replicates of the untreated and treated samples, respectively. Of these, 154 and 150 carbonylated proteins were identified in the three replicates of the untreated and treated samples, respectively (**Tables S1**, **S2**). One hundred and fifteen (115) of the carbonylated proteins were simultaneously found in both the untreated and treated samples, whereas 39 and 35 carbonylated proteins were specifically identified in the untreated and treated samples, respectively (**Figure 2**; **Tables 1**, **2**, **S3**).

**Table 1:** Examples of carbonylated proteins identified only in the control plant samples^a^.

AGI code	Protein name	Accession number	Molecular mass (kDa)	Localization	Peptide sequence	m/z	Charge	% coverage
**A. Protein folding**								
AT2G16600	Peptidyl-prolyl cis-trans isomerase (CYP19-1)	Q38900	18.49	Cytosol	(R)IVMELYADTTPETAENFR(A) (K)FMCQGGDFTAGNGTGGESIYGSK(F) (K)HTGPGILSMANAGANTNGSQFFICTEK(T)	1050.50 1171.00 941.780	2 2 3	39
AT4G38740	Peptidyl-prolyl cis-trans isomerase (CYP18-3)	P34790	18.37	Cytosol	(K)GVGGTGKPLHFK(G) (R)VIPNFMCQGGDFTAGNGTGGESIYGSK(F) (K)HTGPGILSMANAGANTNGSQFFICTVK(T) (K)VGSSSGKPTKPVVVADCGQLS(-) (K)HVVFGQVVEGLDVVK(A)	1196.67 2763.23 2792.34 2072.06 1623.90	2 2 3 2 3	51
AT5G42980	Thioredoxin H3 (TRX3)	Q42403	13.11	Unassigned	(R)FIAPVFADLAK(K) (K)EGEIKETVVGAAK(E) (K)HLDVVFFK(V)	1190.67 1329.71 1003.55	2 2 2	20
AT5G49910	Heat shock 70 kDa protein 7 (HSP70-7)	Q9LTX9	77.00	Unassigned	(R)IINEPTAASLAYGFER(K) (R)VVDWLASTFK(K)	1750.89 1164.62	2 2	16
AT5G56030	Heat shock protein 81-2 (HSP81-2)	P55737	80.07	Unassigned	(K)TNNTLTIIDSGIGMTK(A) (K)HFSVEGQLEFK(A) (K)GIVDSEDLPLNISR(E) (K)EGQNDIFYITGESK(K) (R)APFDLFDTK(K)	1677.86 1319.65 1526.79 1599.74 1052.52	2 2 2 2 2	8
**B. Detoxification**								
AT1G19570	Glutathione S-transferase (DHAR1)	Q9FWR4	23.64	Unassigned	(K)AAVGAPDHLGDCPFSQR(A) (K)WVTDSDVIVGILEEK(Y) (K)TPAEFASVGSNIFGTFGTFLK(S) (K)TLFSLDSFEK(T) (K)YVISGWAPK(V) (R)VSAVDLSLAPK(L)	1796.83 1701.89 2190.10 1185.59 1019.55 1098.63	2 2 2 2 2 2	35
AT2G29450	Glutathione S-transferase U5 (GSTU5)	P46421	26.00	Cytosol	(K)LLGIWASPFSR(R) (K)SPLLLALNPIHK(K)	1245.69 1314.80	2 2	10
AT3G61440	Bifunctional L-3-cyanoalanine synthase/cysteine synthase C1 (CYSC1)	Q9S757	39.93	Mitochondrion	(R)SFGAELVLTDPAK(G) (K)EGLMVGISSGANTVAAIR(L)	1346.71 1744.91	2 2	8
**C. Developmental process**								
AT1G18080	Receptor for activated C kinase 1A (RACK1A)	O24456	35.75	Cytosol	(K)DVLSVAFSLDNR(Q) (R)FSPNTLQPTIVSASWDK(T) (R)STLAGHTGYVSTVAVSPDGSLCASGGK(D) (K)DGVVLLWDLAEGK(K) (K)DGVVLLWDLAEGKK(L)	1334.68 1889.95 2578.24 1413.75 1541.85	2 3 2 2 3	21
AT2G21170	Triosephosphate isomerase (TIM)	Q9SKP6	33.35	Plastid	(R)IDISGQNSWVGK(G) (K)GGAFTGEISVEQLK(D) (K)WVILGHSER(R) (K)VASPQQAQEVHVAVR(G) (R)IIYGGSVNGGNSAELAK(E)	1302.66 1434.74 1095.58 1617.86 1648.84	2 2 2 3 2	21
AT2G42600	Phosphosenolpyruvate carboxylase 2 (PPC2)	Q5GM68	109.76	Cytosol	(K)VSEDDKLIEYDALLLDR(F) (R)FLDILQDLHGEDVR(E) (K)NQTVDLVLTAHPTQSVR(R) (R)HSDVLDAITTHLGIGSYK(E) (K)LADLESAPAAVAR(L) (R)GGGPTHLAILSQPPDTIHGQLR(V) (K)DITPDDKQELDEALQR(E)	2006.02 1668.85 1877.99 1925.99 1282.69 2264.20 1884.91	3 3 2 3 2 4 2	12
AT2G43750	Cysteine synthase (OASB)	P47999	41.66	Plastid	(K)SVLVESTSGNTGIGLAFIAASK(G) (K)IHYETTGPEIWEDTR(G) (R)GKIDILVAGIGTGGTITGVGR(F) (K)IDILVAGIGTGGTITGVGR(F) (R)AFGAELVLTEPAK(G) (K)IQGIGAGFVPK(N)	2122.14 1845.86 1954.12 1769.01 1344.73 1085.63	2 2 2 2 2 2	15
**D. Small molecule binding**								
AT1G24020	MLP-like protein 423 (MLP423)	Q93VR4	17.05	Unassigned	(K)FWVALGDGINLFPK(A) (K)TIQVLAGDGNAPGSIR(L) (R)LITYGEGSPLVK(I) (K)TAHEIDDPHVIK(D)	1575.85 1567.83 1275.71 1373.69	2 2 2 2	27
AT1G76030	V-type proton ATPase subunit B1 (VHA-B1)	P11574	54.11	Unassigned	(R)TVSGVAGPLVILDK(V) (K)AVVQVFEGTSGIDNK(F) (R)TYPEEMIQTGISTIDVMNSIAR(G) (R)QIYPPINVLPSLSR(L) (K)FVMQGAYDTR(N) (R)IALTTAEYLAYECGK(H)	1367.80 1562.80 2468.17 1595.91 1186.54 1701.83	2 2 2 2 2 2	13
AT2G26080	Glycine dehydrogenase (decarboxylating) 2 (GLDP2)	O80988	113.78	Mitochondrion	(K)FSGIFDEGLTESQMIEHMSDLASK(N) (K)FSGIFDEGLTESQMIEHMSDLASK(N) (K)LGTAQVQDLPFFDTVK(V) (K)VTCSDATAIFDVAAK(K) (R)IIGVSVDSSGK(Q) (R)VHGLAGVFALGLK(K)	2671.22 1060.58 1777.93 1567.76 1060.58 1280.76	3 2 2 2 2 2	15
AT3G59970	Methylenetetrahydrofolate reductase 1 (MTHFR1)	Q9SE60	66.29	Cytosol	(K)IDHALETIR(S) (R)SNGIQNVLALR(G) (K)SDSPAIGWGGPGGYVYQK(A) (K)SENWVSNTGESDVNAVTWGVFPAK(E) (K)EVIQPTIVDPASFK(V) (K)LQQEWVVPLK(S)	1066.58 1183.67 1837.86 2593.61 1542.83 1238.70	3 2 2 2 2 2	13
AT4G18480	Magnesium-chelatase subunit ChlI-1 (CHLI1)	P16127	46.27	Plastid	(K)INMVDLPLGATEDR(V) (R)FGMHAQVGTVR(D) (R)FILIGSGNPEEGELRPQLLDR(F) (R)VCSELNVDGLR(G)	1542.77 1201.60 2352.25 1260.62	2 3 3 2	10
AT5G62790	1-deoxy-D-xylulose 5-phosphate reductoisomerase (DXR)	Q9XFS9	51.97	Plastid	(R)VVALAAGSNVTLLADQVR(R) (R)HPEAVTVVTGIVGCAGLKPTVAAIEAGK(D) (K)ETLIAGGPFVLPLANK(H) (R)LPILYTMSWPDR(V) (R)AGGTMTGVLSAANEK(A)	1796.02 2744.49 1638.94 1490.76 1405.69	2 3 2 2 2	19
**E. Oxidoreductase activity**								
AT4G21280	Oxygen-evolving enhancer protein 3-1 (PSBQ1)	Q9XFT3	23.87	Plastid	(K)DIINVKPLIDR(K) (R)KAWPYVQNDLR(S) (K)AWPYVQNDLR(S) (R)YDLNTIISSKPK(D) (K)LFDTIDNLDYAAK(K)	1294.76 1388.72 1260.63 1377.75 1497.74	3 2 2 3 2	21
AT4G25130	Peptide methionine sulfoxide reductase A4 (MSR4)	P54150	28.64	Plastid	(K)TEVGYSHGIVHNPSYEDVCTGTTGHNEVVR(V) (R)SGIYYYTDEQER(I)	3313.51 1522.66	4 2	16
ATCG00280	Photosystem II CP43 reaction center protein (PSBC)	P56778	51.87	Plastid	(R)DQETTGFAWWAGNAR(L) (K)ALYFGGVYDTWAPGGGDVR(K) (R)LGANVGSAQGPTGLGK(Y) (R)SPTGEVIFGGETMR(F) (R)SAEYMTHAPLGSLNSVGGVATEINAVNYVSPR(S)	1708.76 1999.95 1425.76 1479.70 3303.63	2 2 2 2 3	20
ATCG00680	Photosystem II CP47 reaction center protein (PSBB)	P56777	56.04	Plastid	(R)AGSMDNGDGIAVGWLGHPVFR(N) (R)MPTFFETFPVVLVDGDGIVR(A) (K)LAFYDYIGNNPAK(G)	2171.02 2238.14 1484.73	3 2 2	8
**F. Transferase activity**								
AT1G16880	ACT domain-containing protein (ACR11)	Q9FZ47	31.30	Plastid	(R)LGALLDTMNALK(N) (K)NLGLNVVK(A) (R)KVEDPELLEAIR(L) (K)ALIKPLQQVLANSLR(Y) (R)SLLFIESADRPGLLVELVK(I)	1258.70 855.52 1410.77 1663.02 2098.20	2 2 3 3 2	16
AT5G19220	Glucose-1-phosphate adenylyltransferase large subunit 1 (ADG2)	P55229	57.68	Plastid	(R)TVASIILGGGAGTR(L) (K)AMAVDTTILGLSK(E) (R)SGITVILK(N)	1271.72 1318.72 829.53	2 2 2	7
ATCG00830	50S ribosomal protein L2 (rpl2-A)	P56791	29.87	Unassigned	(R)GAIIGDTIVSGTEVPIK(M) (K)NCSATVGQVGNVGVNQK(S) (K)KPVTPWGYPALGR(R)	1668.93 1730.84 1440.79	2 2 2	17

a The full list of the proteins can be accessed in the **supplementary Table 1**.

**Table 2:** Examples of carbonylated proteins identified only in H_2_O_2_-treated plant samples^a^.

AGI code	Protein name	Accession number	Molecular mass (kDa)	Localization	Peptides sequence	m/z	Charge	% coverage
**A. Response to external stimulus**								
AT1G37130	Nitrate reductase [NADH] 2 (NIA2)	P11035	102.85	Cytosol	(R)LEPGLNGVVR(S) (K)HPFNSEAPLNR(L) (R)IIIPGFIGGR(M) (K)ESDNFYHFK(D) (R)NLALVNPR(A) (R)FALPVEDMVLGLPVGK(H) (K)VWYVVESAK(E) (R)DIILAYMQNGEYLTPDHGFPVR(I) (K)GEIGIVFEHPTLPGNESGGWMAK(E)	1052.60 1280.63 1041.63 1185.51 895.520 1699.92 1079.57 2548.24 2425.17	2 2 2 2 2 2 2 3 3	8
AT1G80600	Acetylornithine aminotransferase (WIN1)	Q9M8M7	48.83	Plastid/ Mitochondrion	(R)VFFCNSGTEANEAAIK(F) (K)IAAVFVEPIQGEGGIYSATK(E) (R)SACDAAGSLLVFDEVQCGLGR(T) (R)DSGLLILTAGK(G) (R)IVPPLVISEEEIER(A) (K)EYLDCASGIAVNALGHGDPDWLR(A) (K)VIVGTYAR(A)	1756.81 2049.08 2224.03 1086.63 1621.90 2528.18 877.50	2 2 2 2 2 3 2	18
AT2G37220	RNA-binding protein CP29B	Q9ZUU4	30.72	Plastid	(K)EQSFSADLK(L) (R)SSFGSSGSGYGGGGGSGAGSGNR(V) (K)GFGFVTYDSSQEVQNAIK(S) (K)SLDGADLDGR(Q)	1023.49 1905.78 1988.95 1017.47	2 2 3 2	21
AT3G01480	Peptidyl-prolyl cis-trans isomerase CYP38	Q9SSA5	47.98	Plastid	(K)SIIVAGFAESK(K) (R)SDGFVVQTGDPEGPAEGFIDPSTEK(T) (R)TVPLEIMVTGEK(T) (R)IVLDGYNAPVTAGNFVDLVER(H)	1120.61 2578.17 1315.71 2261.17	2 2 2 2	11
AT4G03280	Cytochrome b6-f complex iron-sulfur subunit (PETC)	Q9ZR03	24.37	Plastid	(K)DALGNDVVAAEWLK(T) (K)GDPTYLVVENDK(T) (K)FLPCHGSQYNAQGR(V) (R)GPAPLSLALAHADIDEAGK(V) (K)VLFVPWVETDFR(T)	1499.76 1348.65 1793.77 1844.97 1506.79	2 2 3 3 2	26
AT4G37930	Serine hydroxymethyl- transferase 1 (SHM1)	Q9SZJ5	57.40	Mitochondrion	(R)YYGGNEYIDMAETLCQK(R) (R)LDESTGYIDYDQMEK(S) (K)SATLFRPK(L) (K)LIVAGASAYAR(L) (K)VLEAVHIASNK(N) (R)GFVEEDFAK(V) (K)LRHEVEEFAK(Q) (K)QFPTIGFEK(E) (K)AYQEQVLSNSAK(F) (K)GLELIPSENFTSVSVMQAVGSVMTNK(Y) (K)ISAVSIFFETMPYR(L) (K)AYQEQVLSNSAK(F) (K)LKDFVSAMESSSTIQSEIAK(L)	2053.88 1805.77 918.530 1090.61 1179.66 1040.48 1256.65 1065.55 1336.66 2737.37 1659.84 1336.66 2170.08	2 2 2 2 2 2 2 2 2 3 2 2 3	21
AT4G39260	Glycine-rich RNA-binding protein 8 (RBG8)	Q03251	16.58	Nucleus	(R)CFVGGLAWATNDEDLQR(T) (R)TFSQFGDVIDSK(I) (R)GFGFVTFK(D) (R)GFGFVTFKDEK(A) (R)VITVNEAQSR(G) (R)SGGGGGYSGGGGGGYSGGGGGGYER(R)	1950.89 1342.64 901.47 1273.64 1115.59 2022.80	2 2 2 2 2 2	44
AT5G08280	Porphobilinogen deaminase (HEMC)	Q43316	41.05	Plastid	(R)GSPLALAQAYETR(E) (K)ILSQPLADIGGK(G) (K)TILPCNLPR(E) (K)VQATLLALAGLK(R) (R)AFLETLDGSCR(T) (R)TPIAGYASK(D) (K)DAGQELLSR(A)	1375.71 1210.69 1082.59 1196.75 1267.59 906.48 987.50	2 2 2 2 2 2 2	20
**B. Catabolic process**								
AT1G32470	Glycine cleavage system H protein 3 (GDH3)	Q9LQL0	17.90	Mitochondrion	(K)YANSHEWVK(H) (K)LTESPGLINSSPYEDGWMIK(V)	1132.53 2252.07	2 2	17
AT1G43670	Fructose-1,6-bisphosphatase (CYFBP)	Q9MA79	37.2881	Cytosolic	(K)LIGLAGETNIQGEEQK(K) (K)LIGLAGETNIQGEEQKK(L) (R)TSVLVSEEDEEATFVEPSKR(G) (K)GNIYSVNEGNAQNWDGPTTK(Y) (R)YVGSMVADVHR(T) (R)SPIFLGSYDDVEEIK(A) (R)TLLYGGIFLYPADK(K) (R)TLLYGGIFLYPADKK(S)	1698.88 1826.98 2251.09 2163.98 1232.60 1710.84 1569.85 1697.94	2 3 3 2 3 2 2 2	24
AT3G46970	Alpha-glucan phosphorylase 2 (PHS2)	Q9SD76	95.16	Cytosolic	(K)ANPEADDATEIAGNIVYHAK(Y) (K)QTYYLSMEYLQGR(A) (K)TVAYTNHTVLPEALEK(W) (R)ALTNAIGNLNLQGPYADALR(T) (K)WITDLDLLTGLR(Q) (R)VTGVSIDPTSLFDIQVK(R)	2098.00 1666.77 1784.93 2084.10 1414.78 1817.98	2 2 2 2 2 2	6
**C. Detoxification**								
AT5G06290	2-Cys peroxiredoxin BAS1-like (2-Cys Prx B)	Q9C5R8	29.78	Plastid	(K)SGGLGDLNYPLVSDITK(S) (K)SFGVLIPDQGIALR(G) (K)EGVIQHSTINNLGIGR(S)	1747.90 1484.84 1706.91	2 2 3	17
AT5G43940	S-(hydroxymethyl) glutathione dehydrogenase (HOT5)	Q96533	40.70	Cytosol	(K)AAVAYEPNKPLVIEDVQVAPPQAGEVR(I) (K)KFGVNEFVNPK(D) (K)FGVNEFVNPK(D) (K)GTAFGGFK(S) (K)VDEYITHNLTLGEINK(A) (K)VCLLGCGVPTGLGAVWNTAK(V) (K)VEPGSNVAIFGLGTVGLAVAEGAK(T) (R)IIGIDIDSK(K) (K)ILYTALCHTDAYTWSGK(D)	2859.51 1277.68 1149.58 783.39 1857.95 2072.06 2255.22 972.55 1998.95	3 3 2 2 3 2 2 2 3	21
**D. Anatomical structure development**								
AT4G29350	Profilin-2 (PRO2)	Q42418	13.9978	Cytosol	(K)DFEEAGHLAPTGLFLGGEK(Y) (K)YMVVQGEAGAVIR(G)	1986.97 1407.72	3 2	24
AT5G24300	Starch synthase (SS1)	Q9FNF2	72.10	Plastid	(K)SYHRPGNPYGDSK(G) (R)ITAGCDILLMPSR(F) (R)FEPCGLNQLYAMR(Y) (R)YGTIPVVHGTGGLR(D) (R)GWVGFNVPISHR(I) (K)TGGLGDVCGSLPIALAGR(G)	1476.68 1461.73 1613.73 1425.77 1367.71 1712.89	2 2 2 2 3 2	8
E. **Structural constituent of ribosome**								
AT1G05190	50S ribosomal protein L6 (RPL6)	O23049	24.71	Plastid	(K)GPLGELALTYPR(E) (R)EVELTKEESGFLR(V) (R)ANQMHGLFR(T) (R)TLTDNMVVGVSK(G) (K)LILVGVGYR(A) (R)ITVSGYDK(S) (K)SEIGQFAATVR(K) (K)ELVLNLGFSHPVK(M)	1285.70 1535.78 1088.52 1262.66 988.61 881.45 1177.61 1451.81	2 3 2 2 2 2 2 2	33
AT2G27710	60S acidic ribosomal protein P2-2 (RPP2B)	Q9SLF7	11.44	Cytosol	(K)GKDLAELIAAGR(E) (K)DLAELIAAGR(E) (K)LASVPSGGGGGVAVASATSGGGGGG GASAAESK(K) (K)VVAAYLLAVLSGK(A)	1212.68 1027.57 2587.25 1302.79	2 2 3 2	39
AT3G27830	50S ribosomal proteinL12-1 (RPL12A)	P36210	20.08	Plastid	(K)IGSEISSLTLEEAR(I) (R)ALTSLALK(E) (K)ELIEGLPK(K)	1503.78 815.51 897.52	2 2 2	16
AT5G27850	60S ribosomal protein L18-3 (RPL18C)	Q940B0	20.97	Cytosol	(R)SNSNFNAVILK(R) (R)APLGQNTVLLR(G) (K)HFGPAPGVPHSNTKPYVR(H) (K)IAVLVGTITDDLR(V)	1205.64 1180.69 1960.01 1384.79	2 2 3 2	21
**F. Lyase activity**								
AT2G05710	Aconitate hydratase 3 (ACO3)	Q9SIB9	108.20	Mitochondrion	(R)ILLESAIR(N) (K)TSLAPGSGVVTK(Y) (R)SNLVGMGIIPLCFK(S) (K)SGEDADTLGLTGHER(Y)	913.56 1115.62 1563.82 1556.71	2 2 2 2	6
AT5G11670	NADP-dependent malic enzyme 2 (NADP-ME2)	Q9LYG3	64.41	Cytosol	(R)DAHYLTGLLPPVILSQDVQER(K) (R)ILGLGDLGCQGMGIPVGK(L) (K)DLIGAVNAIKPTVLIGTSGVGQTFTK(E) (R)AIFGSGSPFDPVVYDGK(T) (K)TYDLGLASNLPR(A) (K)FAESSMYSPVYR(N)	2363.23 1783.94 2599.46 1754.85 1318.69 1435.65	3 2 3 2 2 2	13
AT5G38430	Ribulose bisphosphate carboxylase small chain 1B (RBCS-1B)	P10796	20.29	Plastid	(R)QVQCISFIAYKPPSFTDA(-) (K)FETLSYLPDLTDVELAK(E)	2071.01 1953.00	2 2	49
**G. Carbohydrate derivative binding**								
AT1G19920	ATP sulfurylase 2 (APS2)	Q43870	53.64	Plastid	(R)EDEYLQSLHFNSLR(L) (K)NPVLLLHPLGGFTK(A) (K)EFLFISGTK(M)	1749.84 1504.88 1040.56	2 2 2	10
AT3G22890	ATP sulfurylase 1 (APS1)	Q9LIK9	51.46	Plastid	(R)LDDGSVVNMSVPIVLAIDDEQK(A) (R)VALFNSDGNPVAILSDIEIYK(H) (R)INAGANFYIVGR(D) (R)NPVHNGHALLMTDTR(R) (K)NPILLLHPLGGFTK(A)	2356.18 2277.19 1293.68 1690.82 1518.89	2 2 2 3 2	12
AT4G09320	Nucleoside diphosphate kinase 1 (NDK1)	P39207	16.50	Unassigned	(R)GLIGEVICR(F) (R)KIIGATNPAASEPGTIR(G) (K)IIGATNPAASEPGTIR(G) (R)GDFAIDIGR(N) (R)NVIHGSDSVESAR(K)	1015.55 1694.93 1566.84 962.48 1369.66	2 2 2 2 2	32

a The full list of the proteins can be accessed in the **supplementary Table 2**.

### Exogenous H_2_O_2_ mostly targets proteins involved in defense-related processes for carbonylation

**Figure 3:**
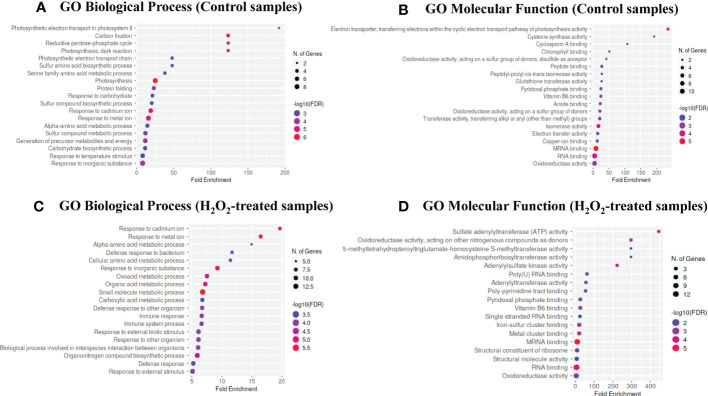
Gene ontology enrichment analysis. The bubble chart of the top 20 Gene Ontology (GO)-terms represented by the carbonylated proteins in the control plant samples (T0) and H_2_O_2_-treated plant samples (T20) were generated using the ShinyGO v0.76 web-based bioinformatics resource. **(A–C)** biological processes. **(B–D)** molecular function. The x-axis indicates fold enrichment. The y-axis indicates GO terms.

To determine the biological processes and molecular functions overrepresented in the lists of carbonylated proteins in response to exogenous H_2_O_2_, we performed a gene ontology (GO) enrichment analysis by using the ShinyGO v0.76 online tool. The carbonylated proteomes derived from the untreated plants and the H_2_O_2_-treated plants were found to be enriched in different biological processes (**Figure 3**). Notably, the carbonylated proteins identified in the samples of untreated plants were enriched in the processes of photosynthesis (GO: 0015979) and generation of metabolic precursors and energy (GO: 0006091), whereas, the carbonylated proteins identified in the samples of H_2_O_2_-treated plants were distinctively enriched in processes related to the immune system response including defense response to other organism (GO: 0051707), interspecies interaction between organisms (GO: 0044419), and external biotic stimulus (GO: 0043207). Concerning the molecular functions, the carbonylated proteins in the control samples were mostly related to the electron transporter, transferring electrons within the cyclic electron transport pathway of photosynthesis activity (GO: 0045156), cysteine synthase activity (GO: 0004124), Cyclosporin A binding (GO: 0016018), chlorophyll binding (GO: 0016168), and oxidoreductase activity acting on a sulfur group of donors, disulfide as acceptor (GO: 0016671). In contrast, the carbonylated proteins in the H_2_O_2_-treated samples were mostly related to amidophosphoribosyltransferase activity (GO: 0004044), sulfate adenylyltransferase (ATP) activity (GO: 0004781), adenylylsulfate kinase activity (GO: 0004020), poly-pyrimidine tract binding (GO: 0008187), and oxidoreductase activity acting on other nitrogenous compounds as donors (GO: 0016661).

**Figure 4:**
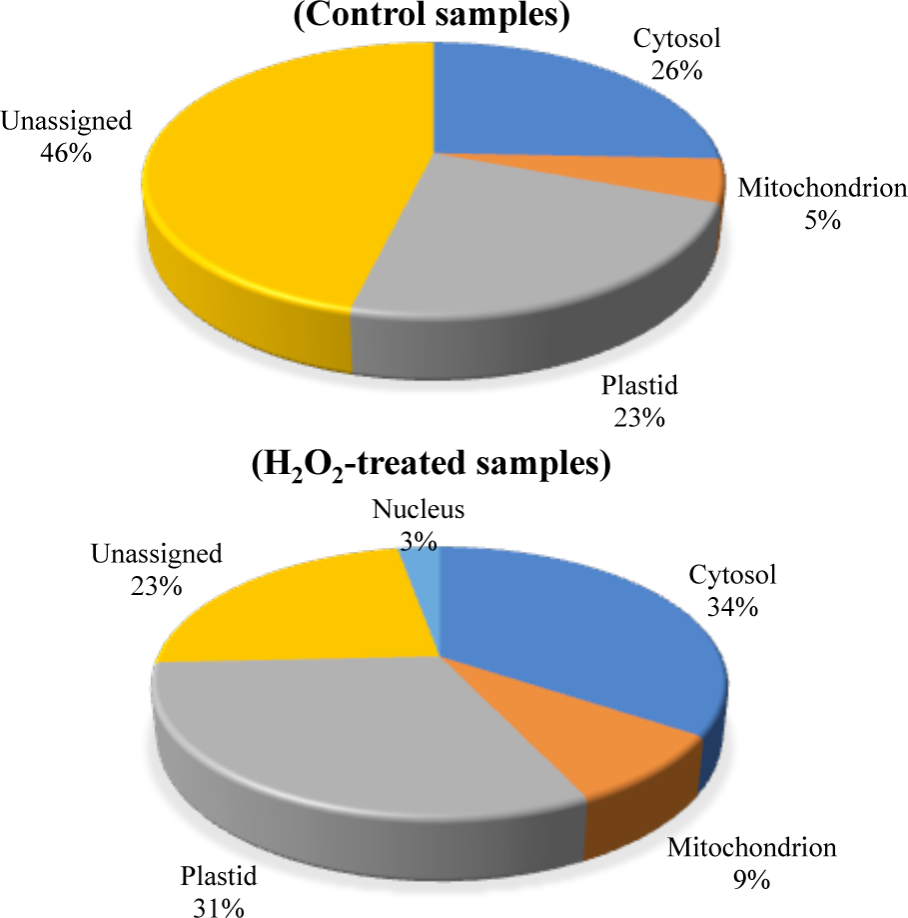
Predicted sub-cellular localization of the carbonylated proteins. The subcellular localization of the carbonylated proteins in the control samples and the H_2_O_2_-treated samples were compared by using the Subcellular Proteomic Database SUBA. **(A)** control plant samples. **(B)** H_2_O_2_-treated plant samples.

We also determined the subcellular localization of the carbonylated proteins identified in the two groups of samples by using the Subcellular localization database for Arabidopsis proteins SUBA (https://suba.plantenergy.uwa.edu.au; Hooper et al., 2017; **Figure 4**). The carbonylated proteins found in the control samples were assigned in the cytosol (26%), plastids (23%), mitochondria (5%), and a significant proportion of proteins with unassigned localization (46%). In comparison, the proportion of unassigned carbonylated proteins was relatively low (23%) in the samples of H_2_O_2_-treated plants, and 2% of the carbonylated proteins in the samples of H_2_O_2_-treated plants were assigned in the nucleus (2%).

### H_2_O_2_-induced protein carbonylation targets the interaction between proteins of the chloroplast redox systems, sulfur metabolism, and translation

**Figure 5:**
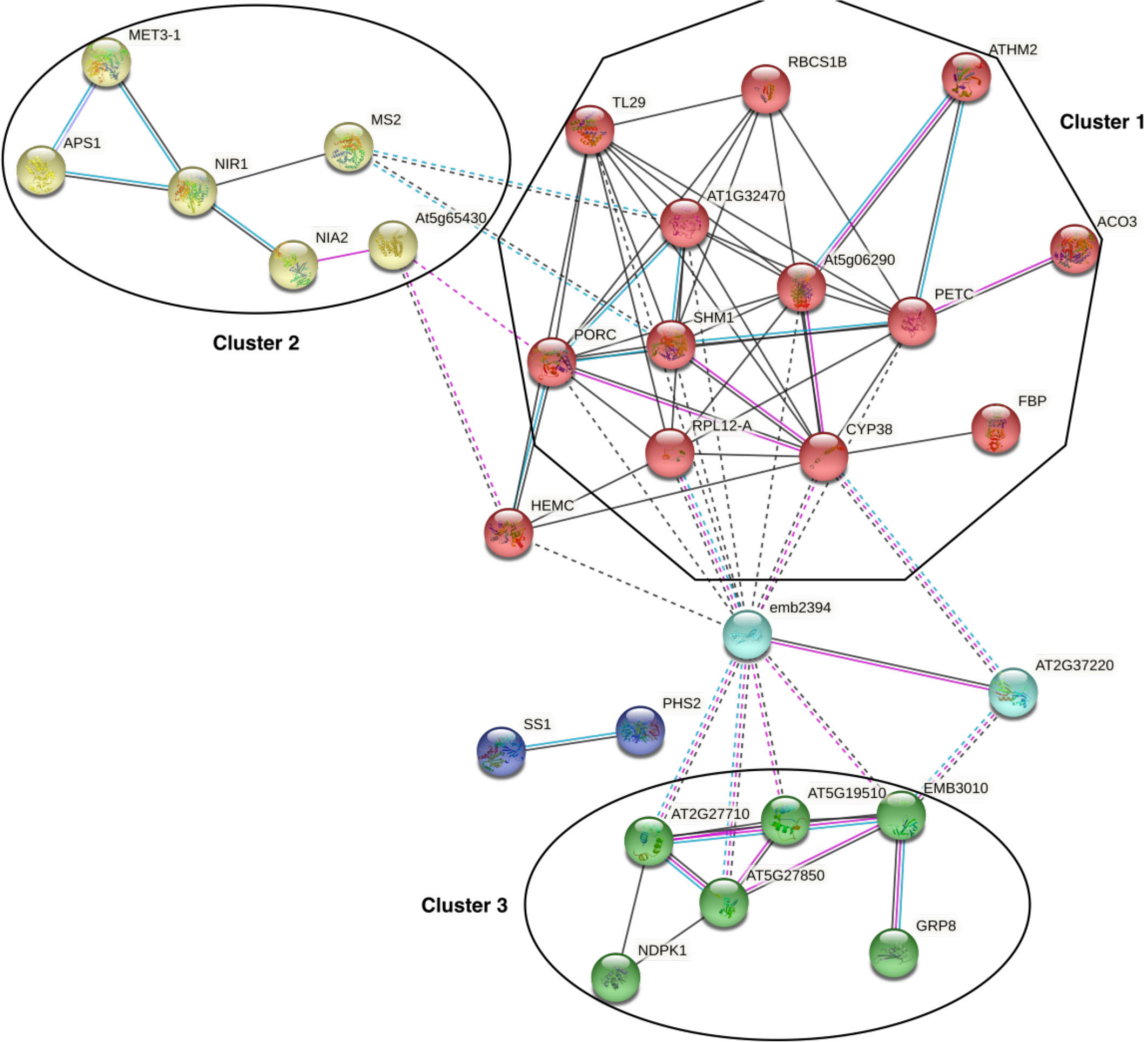
Protein-protein interaction networks infered from the carbonylated proteins. The network was generated from the STRING server by projecting the list of the H_2_O_2_-induced carbonylated proteins onto A. thaliana protein-protein interaction (PPI) data and by using only experimentally determined interactions, curated databases, and coexpression data with default settings. PPI enrichment p-value: < 1.0e-16.

Because carbonylation can affect physical interactions between individual proteins we next probed the extent to which the carbonylated proteome data mapped onto *A. thaliana* protein-protein interaction (PPI) data. A functional network analysis was performed on the carbonylated proteins induced by H_2_O_2_ on the STRING server (Szklarczyk et al., 2021). We limited the sources of information to build the network to only experimentally determined interactions, curated databases, and coexpression data. After removing disconnected nodes, 3 major clusters (PPI enrichment p-value: < 1.0e-16) were revealed within the query list of proteins *via* the MCL clustering algorithm (**Figure 5**). The cluster 1 is dominated by chloroplast proteins with oxidoreductase activity and involved in photosynthesis, defense to bacterium and oxidation-reduction processes. The cluster 2 contains proteins mostly related to sulfur metabolism. This cluster also contains the 14-3-3 PROTEIN G-BOX FACTOR14 KAPPA (GRF8), a 14-3-3 family protein associated with a DNA binding complex that binds to the G box, a well-characterized cis-acting DNA regulatory element found in plant genes. GRF8 was shown to be involved in the regulation of nutrient metabolism and acting as a negative regulator of freezing tolerance that modulates cold-responsive C-repeat-binding factors (CBF) DREB1A AND DREB1B proteins stability by facilitating their ubiquitin-mediated degradation. The cluster 3 was dominated by cytosolic proteins involved in mRNA binding and protein translation. Changing the default settings to add up to 50 interacting proteins did not generate additional cluster, but merely increased the number of genes in the cluster 3 with ribosome proteins. This underscored the potential impact of H_2_O_2_-induced protein carbonylation on the chloroplast redox systems, the sulfur metabolism, translation, and protein homeostasis.

## Discussion

Protein carbonylation is one of the post-translational modifications (PTM) induced by H_2_O_2_ and reactive electrophile aldehydes and ketones, but the biological processes that are targeted are less defined. Whether it contributes to the intracellular signaling by H_2_O_2_ also remained to be clarified further. In this study, we investigated proteins of which activity could be affected by carbonylation induced by exogenous H_2_O_2_ in *A. thaliana* plants. Like protein carbonylation, protein sulphenylation is also induced by H_2_O_2_ under stress conditions and occurs on reactive cysteines (Huang et al., 2019; Yu et al., 2021). The activity of numerous ROS-generating enzymes and signaling proteins was shown to be controlled by sulphenylation (Waszczak et al., 2014; Waszczak et al., 2015), suggesting that protein carbonylation may also control the activity of certain proteins for physiological purposes. In this study, we sprayed the leaves of 21-day-old plants with solutions of 1 and 20 mM of H_2_O_2_ and examined the proteins that became carbonylated thereafter in the leaves. Similar or higher concentrations of H_2_O_2_ were previously used to identify the genes regulated by H_2_O_2_ in transcriptome studies (Dietz et al., 2006; Gadjev et al., 2006). The levels of H_2_O_2_, MDA, and catalase activity that we quantified from the leaves confirmed that the concentration of 20 mM H_2_O_2_ was sufficient to induce MDA and H_2_O_2_ formation *in vivo*, both of which can lead to protein carbonylation in *A. thaliana* (Noctor et al., 2015; Missihoun and Kotchoni, 2017; Morales and Munné-Bosch, 2019; Ciacka et al., 2020). Catalase is an enzyme involved in the detoxification of H_2_O_2_ (Dietz et al., 2006; Gadjev et al., 2006), and MDA is known to be an unconditional marker of stress (Missihoun and Kotchoni, 2017; Morales and Munné-Bosch, 2019). At a concentration of 1 mM, exogenous H_2_O_2_ did not affect the level of carbonylation of proteins in the leaves, whereas at 20 mM, it increased the fluorescence signal of labeled carbonylated proteins. Moreover, we observed that the level of carbonylation of certain proteins was greater in the samples treated with H_2_O_2_ compared to the control samples (**Figure 1**). This indicated that the application of H_2_O_2_ could lead to selective carbonylation of some proteins, certainly those bearing hyperreactive cysteines (Weerapana et al., 2010). Our approach was qualitative, and therefore, does not allow to state which proteins changed in abundance upon the treatment. Detection of modified proteins in our analyses may be driven in part by abundance in the proteome, and peptides from high-abundance proteins could be more easily detected by LC-MS/MS than those from low-abundance proteins. A few carbonylated proteins identified in previous studies in response to salt stress or ABA were also found in this study. These include the Glycine-rich RNA-binding protein 8 (AT4G39260) (Mano et al., 2014), and ASCORBATE PEROXIDASE4 (APX4 or TL29, AT4G09010) and 2-cys-peroxiredoxin BAS1-like 2 (2-Cys PRXB, AT5G06290) (Jaballi and Missihoun, 2022). Because salt stress induces oxidative stress and ABA and there is a crosstalk between ABA and H_2_O_2_ signaling pathways, it is likely that these three proteins are at the crossroads of the plant’s response pathways to ABA and H_2_O_2_. For what we know, APX4 encodes a thylakoid lumen protein associated with the photosystem II but with no proved ascorbate peroxidase activity (Granlund et al., 2009). It was shown to regulate seed vigor and seedling growth in *A. thaliana* (Wang et al., 2014). 2-Cys PRXs are plastid proteins encoded by two genes (2-Cys PRXA and 2-Cys PRXB) in *A. thaliana*. They were recently shown to be part of a redox-sensitive module including cyclophilin 20-3 and cysteine synthase, which integrates sulfur metabolism and oxylipin signaling in the high light acclimation responses (Müller et al., 2017). The 2-Cys PRX would specifically control photosynthesis, sugar, and amino acid metabolism (Müller et al., 2017). The 2-Cys PRX were also shown to be involved in light-dark redox homeostasis and signaling (Dangoor et al., 2012; Yoshida et al., 2018). In leaves, these abundant PRXs represent ~1% of chloroplast proteins and possess diverse activities. Depending on their oxidation and oligomerization states, peroxidase activity, chaperone activity, and transmitter of redox signals were attributed to the two chloroplast 2-Cys PRX in *A. thaliana* (D’Autréaux and Toledano, 2007; Poole et al., 2011; Cerveau et al., 2016; Liebthal et al., 2018). S-nitrosylation, phosphorylation, Cys-glutathionylation, and Lys-acetylation are previously reported PTMs of PRXs, which, in turn, alter PRX properties (Liebthal et al., 2018). The biological importance of the PRXs carbonylation remains to be elucidated. We looked for the information about the residues involved in the carbonylation of these proteins but with limited success. The information was obtained for only a limited number of proteins except the 2-Cys PRXs (**Supplementary Table 4**). Besides these proteins, our results indicated that exogenous H_2_O_2_ triggered the carbonylation of a set of proteins and enzymes involved in defense response, chloroplast redox systems, sulfur and protein metabolism (**Tables 1** and **2**). AtGRP8 (RBG8) is one of these proteins and is located in the nucleus. GRP8 is a glycine-rich RNA-binding protein that plays a role in RNA processing during stress (Streitner et al., 2012). It is involved in mRNA alternative splicing of numerous targets by modulating splice site selection. Its carbonylation in response to H_2_O_2_ suggests that it has a carbonylation-sensitive residue, and we speculate that the carbonylation of AtGRP8 might serve as a node of transcriptional regulation by H2O2. A targeted proteomics approach on the proteins identified in this study will likely be more meaningful in elucidating the biological importance of their carbonylation. Overall, this study showed that exogenous H_2_O_2_, from a certain threshold, (i) increased the intracellular levels of H_2_O_2_ and MDA, and concomitantly, (ii) induced the carbonylation of certain proteins. H_2_O_2_ can activate in one case the vital physiological mechanisms linked to the growth and development of the plant, and in the other case, it can trigger processes leading to the death of the cell and even of the plant (Černý et al., 2018). The role that carbonylated proteins may play in either case in plant tissues remains poorly understood. Although the analysis showed different carbonylated proteins in each sample, it remains to demonstrate the impact of this change on these proteins and the plant. This will be the subject of our investigations in the future. The identification and experimental validation of the sites of carbonylation are required to investigate the consequence of the carbonylation on the protein function. The H_2_O_2_-induced carbonylated proteins that we identified in this study constitute a list of candidate proteins for future investigations about the importance of protein carbonylation in H_2_O_2_ signaling and hormesis effects.

## Data availability statement

The data presented in the study are deposited in the ProteomeXchange repository, accession number PXD037515. This is the link: http://proteomecentral.proteomexchange.org/cgi/GetDataset?ID=PXD037515.

## Author contributions

TM and GF-Y designed the research. GF-Y performed the experiments. GF-Y, AT, and TM analyzed the data and wrote the manuscript. All authors contributed to the article and approved the submitted version.

## Funding

This research was funded through the Natural Sciences and Engineering Research Council of Canada (NSERC) Discovery Grant Program, grant number DGECR-2019-00304 to Tagnon D. Missihoun.

## Acknowledgments

Georges Y. Fangue-Yapseu and Tola Adesola Julius thanks UQTR for the financial support through the UQTR Foundation scholarships.

## Conflict of interest

The authors declare that the research was conducted in the absence of any commercial or financial relationships that could be construed as a potential conflict of interest.

## Publisher’s note

All claims expressed in this article are solely those of the authors and do not necessarily represent those of their affiliated organizations, or those of the publisher, the editors and the reviewers. Any product that may be evaluated in this article, or claim that may be made by its manufacturer, is not guaranteed or endorsed by the publisher.
