# Supplementary material for: Proteome-wide analysis of hydrogen peroxide-induced protein carbonylation in Arabidopsis thaliana
Source: Front Plant Sci. 2022 Dec 5;13:1049681. doi: 10.3389/fpls.2022.1049681 (PMC9760910; doi:10.3389/fpls.2022.1049681)
Supplement: Supplementary file 1 [file DataSheet_1.zip › Table 4.docx]

**Supplementary Table 4.** List of modifications and peptide sequences

| **Tair code** | **Protein name** | **Accession number** | **Peptide sequence*** | **Sites** | **Type of modification** |
| --- | --- | --- | --- | --- | --- |
| ATCG00490 | Ribulose bisphosphate carboxylase large chain | O03042 | (R)ELGVPIVMHDYLTGGFTANTSLSHYCR(D) | H267 | Cinnamaldehyde |
|  |  |  | (R)ELGVPIVMHDYLTGGFTANTSLSHYCR(D) | H267 | β-cyclocitral |
|  |  |  | (R)ELGVPIVMHDYLTGGFTANTSLSHYCR(D) | H267 | 4-Hydroxy-2-hexenal |
| AT4G21640 | Subtilisin-like protease SBT3.15 | F4JJL8 | (R)ILKLKTTR(I) | K120 | 4-Hydroxy-2-hexenal |
| AT3G46810 | Cysteine/Histidine-rich C1 domain family protein | Q9STF5 | (K)LCEACVSQINTDPFYSCELCGFILHQTCANMPRK(K) | C403 | Malondialdehyde (MDA) |
| AT3G13235 | Ubiquitin family protein | F4JC86 | (K)HQCTIDLKENVMTVGGGEVSVPFLQEKDIPSR(F) | H295 | β-cyclocitral |
| AT1G54070 | Dormancy-associated protein homolog 4 | F4HV65 | (R)SIMVTKGNNNVR(G) | K53 | Acrolein |
| AT3G04110 | Glutamate receptor | A0A1I9LLK1 | (R)VLVTAGNK(V) | K432 | Acrolein |

*Putative modified amino acid residue is highlighted in blue.
