# Supplementary material for: Proteome-wide analysis of hydrogen peroxide-induced protein carbonylation in Arabidopsis thaliana
Source: Front Plant Sci. 2022 Dec 5;13:1049681. doi: 10.3389/fpls.2022.1049681 (PMC9760910; doi:10.3389/fpls.2022.1049681)
Supplement: Supplementary file 2 [file DataSheet_2.docx]

Supplementary Material

# Supplementary Figures

Figure S1. Exogenous H_2_O_2_ modulates protein carbonylation in *A. thaliana* plant leaves. (A) Representative gel pictures of carbonylated proteins (a) and the total proteins stained with AzureRed (b) are shown for three batches of samples corresponding to three experimental repetitions. A total of 10 µg of proteins labeled with the carbonyl-reactive probes Cy7.5-Hydrazide probe was loaded in each lane. T0: control, distilled water; T1: H_2_O_2_ at 1 mM; T20: H_2_O_2_ at 20 mM. The bar plot (c) represents the relative fluorescence intensity of carbonylated proteins measured from the gel picture (a) by using the Azure Biosystems software. Results are from at least three independent experiments and represent means ± SEM (n=3). Asterisks denote statistical significance at p < 0.001, one-way ANOVA, Tukey post hoc test. ns: not significant.

Figure S2. Two probes-based gel comparison of carbonylated protein profiles. Representative gel pictures for the carbonylated proteins (a) and the total proteins stained with AzureRed (b) are shown. T0: control, distilled water; T1: H_2_O_2_ at 1 mM; T20: H_2_O_2_ at 20 mM. Carbonylated proteins in the control sample were labeled with a Cy5.5-Hydrazide probe, and those in the H_2_O_2_-treated plant samples were labeled with a Cy7.5- Hydrazide probe. Lanes T0+T1, and T0+T20 combine each equal amount (5 µg) of the Cy5.5-Hydrazide-labeled control and Cy7.5-Hydrazide-labeled H_2_O_2_-treated samples.

Figure S3. Exogenous H_2_O_2_ induces protein carbonylation in *A. thaliana* leaves in a concentration-dependent manner. (A) Representative gel pictures for carbonylated proteins (a) and the total proteins stained with AzureRed (b) are shown. Treatments were as follows: T0: control; T1: 1 mM H_2_O_2_; T5: 5 mM H_2_O_2_; T10: 10 mM H_2_O_2_; T20: 20 mM H_2_O_2_; T50: 50 mM H_2_O_2_. (B) Relative fluorescence intensity of carbonylated proteins measured from the gel picture (a) in (A) by using the Azure Biosystems software. (C) Malondialdehyde (MDA) content of leaves determined using the absorbance of the TBA-MDA (thiobarbituric acid-malondialdehyde) adduct measured at 532 nm. Results are from three independent experiments and represent means ± SEM (n=3). Asterisks denote statistical significance at p < 0.001, one-way ANOVA, Tukey post hoc test. ns: not significant.

**
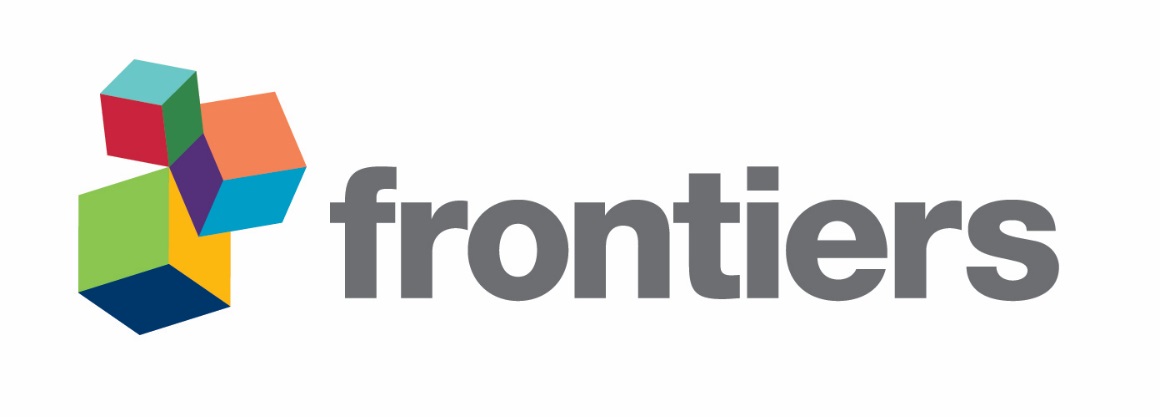
**
